# Data from a fingerprint matching task with experts, trained students and untrained novices

**DOI:** 10.1016/j.dib.2016.09.022

**Published:** 2016-09-23

**Authors:** Sarah V. Stevenage, Christy Pitfield

**Affiliations:** Department of Psychology, University of Southampton, UK

**Keywords:** Fingerprint analysis, Expertise

## Abstract

The data described here provide standard performance measures following administration of a fingerprint matching task to expert analysts, trained students and novice control participants. Measures include accuracy on ‘same’ and ‘different’ trials, and the associated measures of sensitivity of discrimination (d′) and response bias (C). In addition, speed of correct response is provided. The provision of these data will enable the interested reader to conduct meta-analyses relating to questions of fingerprint expertise and fingerprint training (see “Fact or friction: examination of the transparency, reliability and sufficiency of the ACE-V method of fingerprint analysis” (Stevenage and Pitfield, in press) [1]).

**Specifications Table**

**Table:** 

Subject area	Psychology
More specific subject area	Fingerprint Analysis
Type of data	Table, Figure, Excel file
How data were acquired	Computer-based assessment of performance on a fingerprint matching task.
	All stimuli were presented, and all data were recorded using SuperLab 4.5 running on a DELL laptop PC with a 17″ colour monitor and a screen resolution of 1024×768 pixels.
Data format	Analysed
Experimental factors	Three groups of participants were varied, representing established experts (n=12), a trained student group (n=28), and a novice student group (n=26).
	The experts’ description of their process was used as a basis for the training for the student group.
	Accuracy and speed of performance were recorded on a same/different task for all three groups.
Experimental features	A computer-based fingerprint matching task was used in which participants viewed two fingerprint images simultaneously and side by side on-screen. The task was to indicate whether the two fingerprint images belonged to the same person or to two different people. The images measured 8 cm×5.3 cm and ‘same’ trials always used two different images of the same fingerprint.
	A measure of proportion-accuracy (0–1) and of response speed for correct decisions (in milliseconds) is made available for ‘same’ trials and for ‘different’ trials, for each of three fingerprint patterns.
Data source location	Southampton, Hampshire, UK
Data accessibility	Data are provided in this article.

**Value of the data**

• The current data detail the performance of experts, trained students and novice students on a fingerprint matching task. As such, they provide rich information regarding expertise effects on the fingerprint matching task.
• The use of a common task, and the inclusion of a control group of novices, provide rigour that will support future explorations of expertise effects.
• The data here provide baseline levels of performance against which different training methods may be evaluated.
• Finally, the data here offer value through the inclusion of a healthy number of ‘same’ and ‘different’ trials within the matching task. This was made possible through the design of an explicit test rather than through the insertion of test trials within a normal caseload. The benefit is seen through the delivery of a dataset on which robust statistical analyses can be conducted.

## 1 Data

The excel file details the accuracy (0–1) and both mean and median speed of correct decisions (milliseconds) for experts, trained students and novice students in a fingerprint matching task. Data are provided separately for ‘same’ trials and ‘different’ trials. Within each trial type, data are provided separately for the three fingerprint patterns: whorls, radial loops, ulnar loops.

## 2 Experimental design, materials and methods

Fingerprint stimuli were drawn from the BioSecure database and consisted of a target set of 36 fingerprints (12 whorls, 12 radial loops and 12 ulnar loops). Each fingerprint was depicted by a high quality image (simulating the image obtained in a custody suite) and by a low quality image (simulating the image that may be found at a crime scene). ‘Same’ trials consisted of the presentation of the high quality image alongside its corresponding low quality image. This ensured that identical images were never presented, and that the task presented a degree of realism. In addition, 18 foil fingerprints were selected (6 whorls, 6 radial loops and 6 ulnar loops) and these were used in the ‘different’ trials. The construction of these ‘different’ trials was such that the fingerprint pattern was held constant across target and foil fingerprint so that the task was not too easy.

Following training as determined by the participant group, all participants completed the same fingerprint matching task which consisted of a practice phase of 8 trials, and an experimental phase of 72 trials. Half of each set represented ‘same’ trials and the other half represented ‘different’ trials, with the 36 target fingerprints being presented in both a ‘same’ trial and a ‘different’ trial for each participant. In both phases, the order of trials was randomised, and the only difference between practice and experimental phases was the inclusion of feedback in the practice phase.

**Fig. 1:**
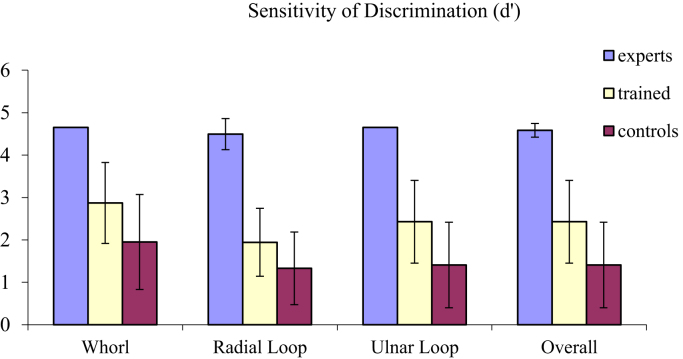
Mean Sensitivity of discrimination (d′) for each of the three fingerprint patterns, and for trials overall, for each of the participant groups (vertical bars represent±1 standard deviation).

**Fig. 2:**
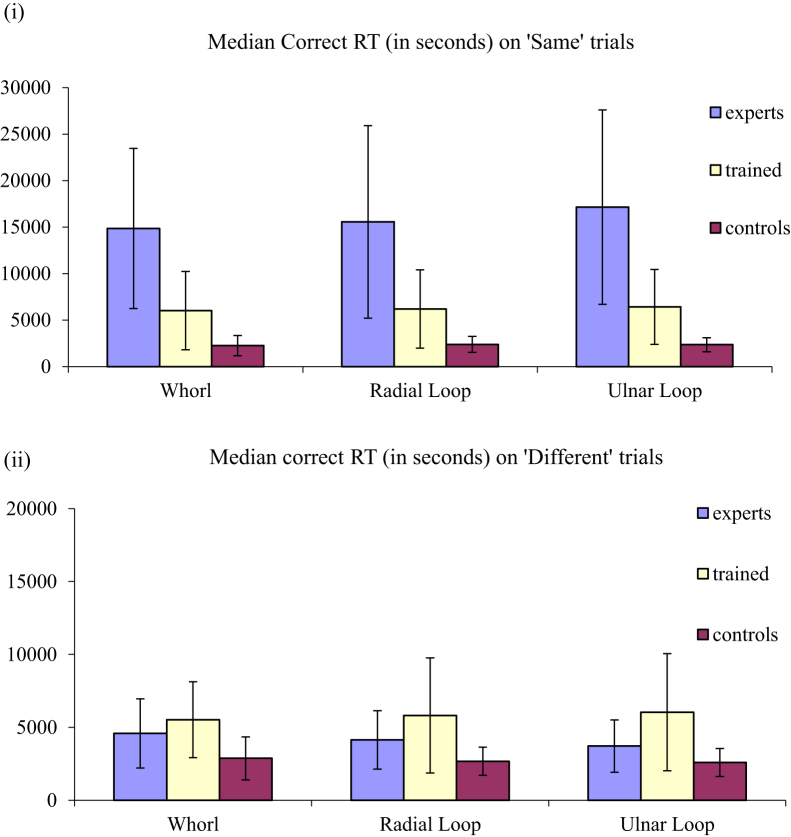
Group average of the median response time of correct decisions on (i) ‘same’ trials, and (ii) ‘different’ trials for each of the three fingerprint patterns, and for each of the participant groups (with error bars showing±1 standard deviation).

In all trials, two fingerprint images were displayed simultaneously and side by side on the computer screen, with the prompt question ‘Same or Different?’ below them. The images remained on screen until participant response, with participants pressing ‘s’ if they considered the pair to come from the SAME individual, and ‘d’ if they considered the pair to come from DIFFERENT individuals. There was no option to provide an ‘inconclusive’ decision. Throughout the task, participants were asked to prioritise accuracy over speed and were reminded of the consequence of inaccurate decisions in the real world. Accuracy (see Table 1 and Fig. 1) and speed of performance (see Fig. 2) were recorded and these data are available in the associated data file. All work reported here was conducted in line with the British Psychological Society ethical standards and ethical requirements of the host institution. Full details of stimuli and procedure are provided in [1].

## Figures and Tables

**Table 1:** Mean Accuracy on ‘Same’ and ‘Different’ trials for each of the three fingerprint patterns, and for trials overall, for each of the participant groups (standard deviation provided in parentheses).

	Experts	Trained students	Novice students
**Whorls**			
Accuracy (‘same’ trials)	1.00 (.00)	.87 (.14)	.85 (.15)
Accuracy (‘different’ trials)	1.00 (.00)	.86 (.16)	.71 (.19)
**Radial Loops**			
Accuracy (‘same’ trials)	.99 (.03)	.85 (.13)	.80 (.14)
Accuracy (‘different’ trials)	1.00 (.00)	.73 (.16)	.61 (.20)
**Ulnar Loops**			
Accuracy (‘same’ trials)	1.00 (.00)	.87 (.14)	.82 (.12)
Accuracy (‘different’ trials)	1.00 (.00)	.79 (.17)	.62 (.19)

**Overall**			
Accuracy (‘same’ trials)	.995 (.01)	.86 (.12)	.82 (.12)
Accuracy (‘different’ trials)	1.00 (.00)	.79 (.15)	.64 (.16)
